# Quantitative Phosphoproteome Analysis of *Clostridioides difficile* Toxin B Treated Human Epithelial Cells

**DOI:** 10.3389/fmicb.2018.03083

**Published:** 2018-12-17

**Authors:** Johannes Junemann, Ingo Just, Ralf Gerhard, Andreas Pich

**Affiliations:** Hannover Medical School, Institute for Toxicology, Hanover, Germany

**Keywords:** *Clostridioides difficile*, phosphoproteome, shotgun proteomics, small GTPases, TcdB

## Abstract

The large clostridial glucosylating toxin B (TcdB) is a major virulence factor of the nosocomial pathogen *Clostridioides difficile*. TcdB inhibits small GTPases by glucosylation leading to impaired downstream signaling. TcdB also possesses a glucosyltransferase independent effect described as pyknosis. To elucidate the impact of TcdB and its glucosylation-inactive mutant TcdB_NXN_ on the kinome of human cells, SILAC labeled HEp-2 cells were treated with 2 nM TcdB for 8 h. Phosphopeptides were enriched using SCX chromatography, IMAC and TiO_2_ followed shotgun mass spectrometry analysis. Overall 4,197 phosphopeptides were identified; more than 1,200 phosphosites responded to treatment with TcdB or TcdB_NXN_. The data suggested that predominantly stress-activated MAPK-dependent signaling pathways were triggered by toxin B treatment.

## Introduction

*Clostridioides difficile* is one of the most common human nosocomial pathogens and causes antibiotic-associated diarrhea leading sometimes to severe pseudomembranous colitis (Loo et al., 2005; Voth and Ballard, 2005). The main virulence factors are *C. difficile* toxin A (TcdA) and toxin B (TcdB) (Just and Gerhard, 2004). They both belong to the clostridial glucosylating toxins (CGT) that inactivate small GTPases by transferring a glucose moiety to a highly conserved threonine residue (Thr-35 in Rho and Thr-37 in Rac/Cdc42) (Just et al., 1995), while TcdB is up to 1,000 times more potent than TcdA (Chaves-Olarte et al., 1997). In case of TcdB Rho, Rac, and Cdc42 are glucosylated causing a perturbed downstream signaling of the affected small GTPases (Kuehne et al., 2010; Zeiser et al., 2013; Genth et al., 2014). This leads initially to a rearrangement of the cytoskeleton and cell cycle arrest (cytopathic effect) (Reinert et al., 2005; Halabi-Cabezon et al., 2008) and finally to apoptosis (cytotoxic effect) (Matarrese et al., 2007). Recently, it has been shown that high concentrations of TcdB, but not TcdA, have an additional effect causing necrotic cell death termed pyknosis (Farrow et al., 2013; Wohlan et al., 2014; Chumbler et al., 2016; Frädrich et al., 2016). This is manifested in morphological changes such as chromatin condensation, ballooning of the plasma membrane and loss of membrane integrity. Interestingly, this effect is also triggered by the glucosyltransferase-deficient mutant TcdB_NXN_ and is therefore independent of small GTPase glucosylation (Wohlan et al., 2014). It has been suggested that ROS production and involvement of the NADPH oxidase complex are responsible for this effect. However, exact mechanisms of both effects – glucosyltransferase-dependent and independent – are still mostly unknown. Recently, several proteome studies have been conducted to investigate the impact on the proteome and to elucidate the affected downstream signaling pathways by TcdA and TcdB treatment with variating toxin concentrations (Zeiser et al., 2013; Jochim et al., 2014; Junemann et al., 2016; Erdmann et al., 2017). Both toxins exhibit similar effects on the proteome of human epithelial cells when using moderate toxin concentrations. Especially proteins associated with cytoskeleton organization, signaling, cell–cell contact and cell proliferation are significantly altered. Interestingly, these protein groups were also affected by inducing pyknosis with high TcdB_NXN_ concentrations, except proteins involved in GTPase-related signaling.

In this study a comprehensive phosphoproteome study was conducted to further investigate the involved signaling pathways which are altered after TcdB and TcdB_NXN_ treatment (Stasyk and Huber, 2012). Pyknosis-inducing conditions and the human epithelial cell line HEp-2 were chosen, in order to cover the glucosyltransferase-independent effect. With the use of SCX chromatography and IMAC and TiO_2_ phosphopeptide enrichment techniques in total more than 1,000 significantly altered phosphosites were identified which alterations shed more light on intracellular regulatory processes upon toxin treatment.

## Materials and Methods

### Cultivation of HEp-2 Cells and SILAC Labeling

The human epithelial cell line HEp-2 was cultured in 75 cm^2^ flasks in a humidified 5% CO_2_ atmosphere at 37°C and 95% humidity. Cells were cultivated in Minimum Essential Media (MEM) without arginine and lysine (Thermo Fisher Scientific, United States). The media was supplemented with 10% dialyzed bovine fetal calf serum (Silantes, Germany), 100 U/ml penicillin, 0.1 mg/ml streptomycin (Merck, Germany) and 0.4 mM L-proline (Sigma, Germany) in order to avoid arginine-proline conversion (Lössner et al., 2011). For the metabolic labeling 0.6/0.4 mM Arg-0/Lys-0 (light), Arg-6/Lys-4 (medium), or Arg-10/Lys-8 (heavy) (Silantes, Germany) were added according to SILAC protocol (Ong et al., 2002). Complete incorporation of stable isotope labeled amino acids was checked prior to experiments by LC-MS. Cells were passaged every 3–4 days at a 1:5 split ratio.

### Treatment of HEp-2 Cells and Sample Preparation

At a confluency of 75% the differently labeled HEp-2 cells were treated with 2 nM TcdB or TcdB_NXN_ for 8 h. Recombinant toxins were generated using the *Bacillus megaterium* expression system as previously described (Olling et al., 2011). Untreated cells cultured in SILAC-media were used as control. All experiments were carried out in triplicates including a label switch. Changes in cell morphology were documented by phase contrast microscopy (Zeiss, Germany). After treatment, cells were washed twice with ice cold PBS and subsequently harvested by scraping cells and dissolved in lysis buffer containing 50 mM ammonium bicarbonate (pH 8.0), 8 M Urea, 1 mM sodium ortho-vanadate, complete EDTA-free protease inhibitor cocktail (Roche) and phosSTOP phosphatase inhibitor cocktail (Roche). Cells were homogenized on ice by sonication and cell debris was removed by centrifugation at 16,000 *g* for 20 min at 4°C and total protein concentrations was determined using a Lowry assay (Bio-Rad). Equal amounts (1.3 mg) of differently labeled and treated lysates were combined as already described (Zeiser et al., 2013; Junemann et al., 2016).

### Protein Digestion and Fractionation by SCX

Proteins were reduced with dithiothreitol (DTT) (5 mM) for 1h at 37°C and subsequently alkylated with iodoacetamide IAA (10 mM) in the dark at room temperature for 30 min. Alkylation was stopped by adding DTT at a final concentration of 5 mM. Lysates were diluted with 50 mM ABC to a final urea concentration below 4 M. Proteins first were digested with Lys-C (Wako) at a 1:150 enzyme/protein ratio for 4 h at 37°C and then trypsin (1:80; Promega) was added followed by an overnight incubation at 37°C. Digestion was stopped by acidification with TFA to a final concentration of 1%. Peptide solution was desalted using Sep-Pak tC18 cartridges (Waters) according to the manufacturer’s protocol.

Dried peptides were dissolved in SCX Buffer A (7 mM KH_2_PO_4_, pH 2.65, 30% ACN) and separated by strong cation exchange (SCX) using an Agilent 1200 HPLC equipped with a PolySULFOETHYL A column (250 mm × 9.4 mm; 5 μm beads, pore size 200Å) (259-SE0502, PolyLC Inc.). Chromatography was performed by increasing SCX Buffer B concentration (7 mM KH_2_PO_4_, 350 mM KCl, pH 2.65, 30% ACN) from 1–30% over 40 min at a flow rate of 2 ml/min. Twelve 5-min fractions were collected over the full run, lyophilized and subsequently desalted using Sep-Pak tC18 cartridges (Waters). Fractions 1/2, 3/4, and 11/12 were pooled for phosphopeptide enrichment resulting in a total of 9 fractions.

### Phosphopeptide Enrichment

A 2D affinity chromatography was conducted for phosphopeptide enrichment for each of the 9 fractions. In the first step peptides were subjected to an immobilized metal affinity chromatography using a Fe-NTA phosphopeptide enrichment kit according to the manufacturer’s protocol (#88300, Thermo Fisher Scientific). Eluted phosphopeptides were acidified by TFA to final concentration of 2.5% and dried by vacuum centrifugation. All flow-troughs after sample loading were pooled, dried by vacuum centrifugation and stored for subsequent enrichment step by metal oxide affinity chromatography (MOAC) using the TiO_2_ phosphopeptide enrichment spin tips (#88303, Thermo Fisher Scientific). Spin tips were equilibrated by washing with Buffer A (80% ACN/0.4% TFA) first and then with Buffer B (57% ACN/0.3% TFA/25% lactic acid). Peptides were suspended in Buffer B and applied to the spin tips. After reapplying samples, spin tips were again washed with Buffer B and three times with Buffer A before peptides were eluted with 1.5% NH_4_OH first and then with 5% pyrrolidine. Eluted samples were acidified with TFA to a final concentration of 1.25% and dried by vacuum centrifugation. All IMAC and TiO_2_ elution fractions were cleaned up prior to MS analysis using graphite spin columns (#88302, Thermo Fisher Scientific) according to the manufacturer’s protocol.

### Liquid Chromatography Mass Spectrometry (LC-MS)

Dried phosphopeptides were reconstituted in 2% ACN/0.1% TFA and analyzed by an Obritrap Velos mass spectrometer connected to an Ultimate 3000 RSLC nanoflow system (Thermo Fisher Scientific). Samples were loaded on a trap column (2 cm length, 75 μm ID, 3 μm C18 particles) at a flow rate of 6 μl/min of 0.1% TFA for 5 min. The trap column was switched in line with the analytical column (Acclaim PepMap, Thermo Fisher Scientific, 50 cm length, 75 μm ID, 2 μm C18 particles,) and peptides were eluted at a flow rate of 250 nl/min and at 45°C column temperature by an increasing multistep linear acetonitril gradient from 4 to 25% in 105 min and from 25 to 50% in the following 35 min. The column outlet was directly connected to the nano electrospray source of the mass spectrometer and peptides were ionized with a spray voltage of 1.35 kV using metal-coated fused silica emitter.

The Orbitrap Velos mass spectrometer was operated in data-dependent acquisition mode recording survey scans in the orbitrap mass analyzer with a mass range from 300 to 1600 at a resolution of 60,000 at m/z 400. The five most intense precursors with a charge state of +2 or higher were selected for CID fragmentation with a normalized collision energy of 38 using multi-stage activation for the neutral loss masses of phosphoric acid and MS/MS spectra were acquired in the linear ion trap mass analyzer. Dynamic exclusion duration was set to 30 s.

### Data Processing

Raw data were processed with MaxQuant software (version 1.5.3.30) (Cox and Mann, 2008) and peptides were identified by searching against all human entries of the UniProtKB/Swiss-Prot database using the Andromeda search engine (Cox et al., 2011). Propionamidation (C) was set as fixed modification and a maximum of two missed cleavages was allowed. Phosphorylation (S/T/Y), oxidation (M), deamidation (N/Q) and acetylation (protein N-terminal) were set as variable modifications. A false discovery rate of 0.01 on peptide and protein level was used for identification and “re-quantify” and “match between runs” options were checked. For quantification a minimum ratio count of 1 was used and peptides had to be identified in at least 2 replicates for statistical analysis. Data were analyzed and visualized with the software tools Perseus (version 1.5.2.6) (Cox and Mann, 2012; Tyanova et al., 2016) and Cytoscape (version 3.4) Shannon et al., 2003). Two sided on-sample Student’s *t*-test was applied for the comparison between TcdB vs. control and TcdB_NXN_ vs. control.

## Results

### Altered Morphology by TcdB and TcdB_NXN_ Treatment

**FIGURE 1:**
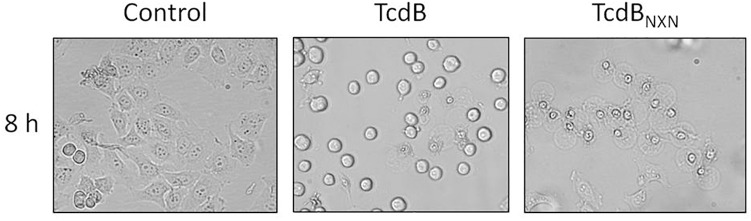
Morphological changes after TcdB or TcdB_NXN_ treatment. Subconfluent HEp-2 cells were treated either with 2 nM TcdB or 2 nM TcdB_NXN_ for 8 h. Control cells were not treated and illustrate normal morphology. TcdB treatment led to typical rounding of most cells and also some pyknotic cells could be observed by phase-contrast microscopy; whereas in consequence of TcdB_NXN_ treatment all cells exhibited only pyknotic morphology, which was manifested by blistering and chromatin condensation.

HEp-2 cells were used to elucidate the impact of TcdB and TcdB_NXN_ on the phosphorylation status of cellular proteins designated as phosphoproteome. According to previous proteome studies cells were treated with a high toxin concentration of 2 nM for 8 h to induce both, cell rounding and the pyknotic phenotype. After TcdB treatment most cells exhibited the typical cell rounding morphology (Figure 1) and about 20% of the cells showed the pyknotic morphology. The glucosyltransferase-deficient mutant TcdB_NXN_ only revealed the pyknotic phenotype for almost all cells. No morphological changes were observed in the control.

### Quantitative Phosphoproteome Analysis After TcdB or TcdB_NXN_ Treatment

**FIGURE 2:**
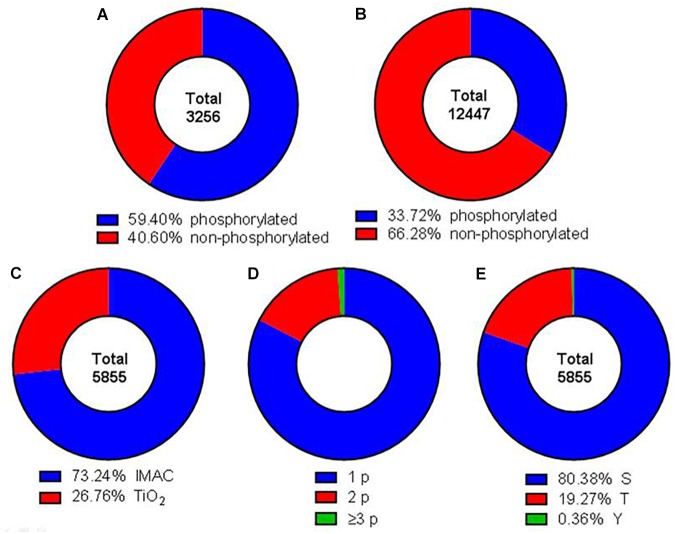
Comprehensive characterization of the obtained dataset. Donut charts illustrating the percentage of identified phosphorylated and non-phosphorylated proteins **(A)** and peptides **(B)**. Red areas represent phosphorylated peptides and proteins, blue areas not phosphorylated proteins and peptides. **(C)** Total phosphopeptides have been enriched using IMAC (blue) or TiO2 (red) techniques. **(D)** Identified phosphopeptides contained either one (blue), two (red), or three or more (green) phosphor groups. **(E)** Phosphorylation was detected at serine (blue), threonine (red), and tyrosine (green) residues.

The phosphoproteome was analyzed by using a SILAC triplex shotgun approach in combination with SCX and phosphopeptide enrichment by IMAC and TiO_2_ prior to MS analysis. With this technique in total 3,256 protein groups were identified by sequence identification of 12,447 peptides (Supplementary Tables 1, 2). Nearly 50% of the identified proteins possessed at least one phosphorylation site (Figure 2A). A phosphorylation site was determined for 5,855 peptides (Figure 2B). Out of these phosphosites 73.2% were identified by IMAC and 26.8% by TiO_2_ enrichment (Figure 2C). Around 82.6% of the identified phosphopeptides were monophosphorylated, while 16.3% were double phosphorylated and about 1% was multiphosphorylated (Figure 2D). About 80% of the phosphate moieties were located at serine residues, 19% at threonine residues and only less than 1% at tyrosine residues (Figure 2E). The analysis of phosphorylation motifs of all regulated phosphosites revealed the consensus sequence R/X-X-S-P and S-P for phosphoserine and T-P for phosphothreonine (data not shown).

**FIGURE 3:**
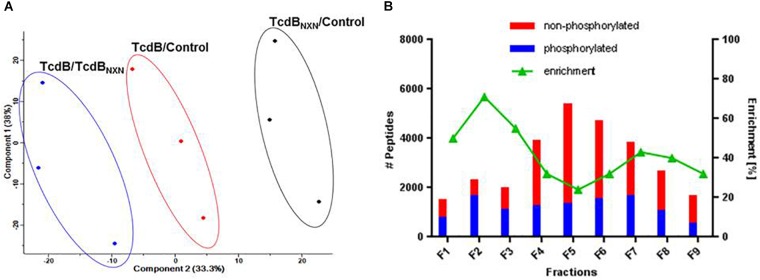
Data reproducibility and phosphopeptide enrichment method analysis. **(A)** Principal component analysis of SILAC labeled replicates illustrating the workflow reproducibility. **(B)** Histogram showing the distribution of identified phosphorylated and non-phosphorylated peptides and the percentage of enrichment across each fraction of SCX chromatography.

The quality of data was supported by a principle component analysis (PCA) of all phosphopeptides identified in the 3 replicates (Figure 3A). Each possible comparison (TcdB/Control; TcdB_NXN_/Control; TcdB/TcdB_NXN_) clustered properly showing the reproducibility of the fractionation and enrichment method.

All SCX fractions contributed to the total quantity of identified phosphopeptides (Figure 3B). Especially in the first three fractions more than 50% of the identified peptides contained a phosphate moiety. In contrast, the middle fractions contained mainly non-phosphorylated peptides, while the amount of phosphorylated peptides remained comparatively constant across all fractions. The number of identified peptides was highest in fraction 5 and declined continuously to fraction 9.

**FIGURE 4:**
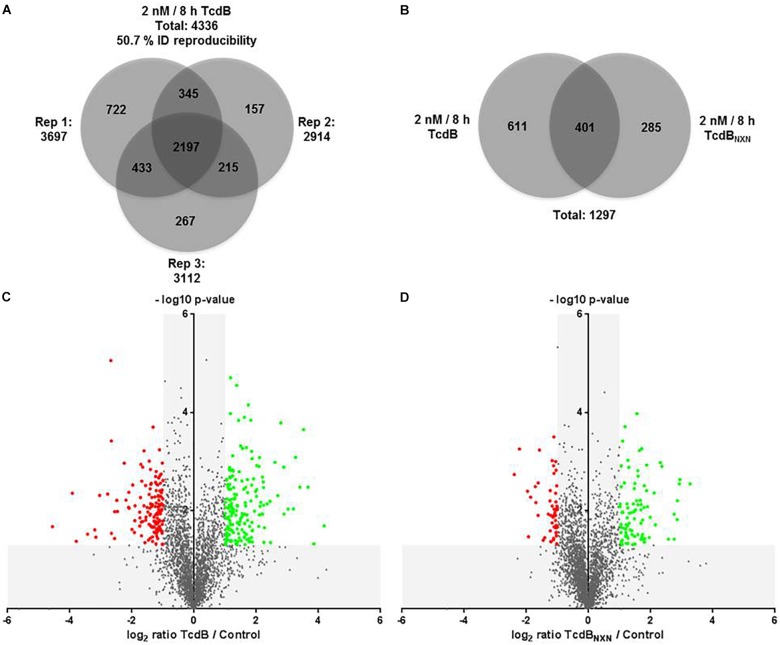
Phosphoproteome analysis of TcdB or TcdB_NXN_ treated HEp-2 cells. **(A)** Venn diagram of all identified phosphosites with a localization probability higher than 75% for all three replicates. **(B)** Venn diagram of all significantly regulated phosphosites as consequence of TcdB or TcdB_NXN_ treatment. Volcano plots of TcdB **(C)** or TcdB_NXN_ **(D)** treated vs. untreated control cells. Negative log_10_ *p*-values were plotted against the log_2_ phosphosite ratios illustrating the significantly regulated sites (red dots: down-regulation; green dots: up-regulation) with a minimum fold change of 2 and a *p*-value less than 0.05.

The comparison of TcdB vs. control revealed in total 4,336 class-I-phosphosites with a localization probability higher than 75%. The number of identified phosphosites was 3,697 for replicate 1, 2,914 for replicate 2 and 3,112 for replicate 3 (Figure 4A). 2,197 phosphosites were identified in all the replicates, resulting in a reproducibility of 50.7%. The comparison of TcdB_NXN_ vs. control resulted in very similar numbers (data not shown). Phosphosites had to be identified in at least two replicates in order to be considered for quantification.

TcdB treatment for 8 h with 2 nM led in total to 1,012 significantly altered phosphosites, whereas TcdB_NXN_ treatment resulted in 686 changed phosphosites. The overlap was 401 phosphosites, while 611 and 285 were altered exclusively by TcdB or TcdB_NXN_, respectively (Figure 4B). When taking only phosphosites with a biological relevance with a fold change of at least 2 into account, 177 up-regulated and 141 down-regulated sites after TcdB treatment were obtained. In case of TcdB_NXN_ 86 phosphosites were up-regulated and 53 down-regulated. It is noticeable that more phosphosites exhibited a stronger phosphorylation than vice versa indicating a general stronger activation of phosphoproteins (Figures 4C,D).

The 10 most up- and down-regulated phosphosites with the corresponding proteins and genes are presented in Table 1 for TcdB and Table 2 for TcdB_NXN_ treatment. It can be noted that the regulation factors were much higher as a consequence of TcdB treatment than after TcdB_NXN_ treatment. The highest up-regulated phosphosite in case of TcdB was S-63 of the transcription factor AP-1 with a fold change (fc) of 18.1, while TcdB_NXN_ treatment led to an up-regulation of phosphosite at S-208 of Serine/arginine-rich splicing factor 2 (fc = 9.6). The most down-regulated phosphosites was S-5448 of neuroblast differentiation-associated protein AHNAK (fc = −23.6) in case of TcdB and a doubly phosphorylated peptide of 40S ribosomal protein S6 (S-236; T-241) with a fold change of −5.2 after TcdB_NXN_ treatment. Interestingly, 4 more phosphorylated peptides of 40S ribosomal protein S6 were highly down-regulated. When looking at TcdB it is noticeable that many GTPase effector or regulation proteins and transcription factors enclose the most strongly regulated phosphopeptides.

**Table 1:** Top 10 up- and down-regulated phosphosites after 8 h treatment with TcdB.

Uniprot ID	Gene name	Protein name	Peptide sequence	log_2_ Ratio TcdB/control	*p*-Value	Amino acid	Peptide score
P05412	JUN	Transcription factor AP-1	NSDLLT**S**PDVGLLK	4.18	2.05E-02	S-63	200.33
M0QZ04	ZFP36	Tristetraprolin	ST**S**LVEGR	3.85	4.77E-02	S-67	123.79
P05412	JUN	Transcription factor AP-1	LA**S**PELER	3.67	3.32E-03	S-73	111.27
Q9NQW6	ANLN	Actin-binding protein anillin	NKA**S**PQSEFMPSK	3.52	2.24E-04	S-792	127.49
Q09666	AHNAK	Neuroblast differentiation-associated protein AHNAK	LK**S**EDGVEGDLGETQSR	3.40	3.32E-03	S-135	255.63
E5RJU9	MTDH	Protein LYRIC	LSSQI**S**AGEEK	3.26	8.28E-04	S-275	117.2
G3V1T9	RBM7	RNA-binding protein 7	SF**S**SPENFQR	3.19	9.30E-03	S-16	125.36
P05412	JUN	Transcription factor AP-1	LQALKEEPQTVP EMPGETPPL**S**PIDMESQER	3.02	9.36E-03	S-243	83.944
K7EMZ9	LSM14A	Protein LSM14 homolog A	TQL**S**QAEVHK	2.89	1.18E-02	S-173	96.135
V9GYM8	ARHGEF2	Rho guanine nucleotide exchange factor 2	SE**S**LESPRGER	2.88	1.31E-03	S-690	145.81
							
Q09666	AHNAK	Neuroblast differentiation-associated protein AHNAK	I**S**APNVDFNLEGPK	−4.56	2.13E-02	S-5448	188.9
Q9H7D0	DOCK5	Dedicator of cytokinesis protein 5	**S**LQLMDNR	−3.92	4.44E-03	S-1766	130.1
Q9H3Q1	CDC42EP4	Cdc42 effector protein 4	AGEPDGE**S**LDEQPSSSSSK	−3.79	4.23E-02	S-64	192.16
Q9H2G2	SLK	STE20-like serine/threonine-protein kinase	RA**S**SDLSIASSEEDK	−3.43	3.03E-02	S-340	228.65
Q5VZK9	LRRC16A	Leucine-rich repeat-containing protein 16A	RS**S**GFISELPSEEGK	−3.20	2.47E-02	S-968	216.75
Q9Y446	PKP3	Plakophilin-3	**T**LQRLSSGFDDIDLPSAVK	−3.16	3.50E-02	T-308	127.41
Q13177	PAK2	Serine/threonine-protein kinase PAK 2	YL**S**FTPPEK	−3.04	4.94E-03	S-141	166.29
Q9Y5K6	CD2AP	CD2-associated protein	**S**VDFDSLTVR	−2.78	4.62E-03	S-458	177.1
Q00587	CDC42EP1	Cdc42 effector protein 1	NAI**S**LPQLNQAAYDSLVVGK	−2.68	8.80E-06	S-121	249.25
P46937	YAP1	Transcriptional coactivator YAP1	ISQ**S**APVK	−2.67	3.81E-04	S-276	112.64

## Discussion

For the first time the effects of TcdB or glucosyltransferase deficient mutant TcdB_NXN_ on the phosphoproteome of target cells were examined. *C. difficile* toxins inhibit small GTPases of the Rho- and Ras-family by glucosylation leading to perturbation of affected signaling pathways and subsequent actin cytoskeleton breakdown. Since protein phosphorylation plays an important role in signaling pathways a phosphoproteome approach has been chosen to reveal further insights into GTPase down-stream processes induced by *C. difficile* toxins (Stasyk and Huber, 2012). A SILAC approach was chosen to precisely quantify the toxin-induced effects in HEp-2 cells which show a clear pyknotic phenotype. Phosphopeptides were enriched by a combination of IMAC and TiO_2_ materials prior to LC-MS shotgun analysis which is an approved strategy to detect as much phosphopeptides as possible.

With the chosen setup 4,197 phosphorylated peptides containing 5,855 phosphosites were identified. Of these phosphosites 4,336 had a localization probability higher than 75% and therefore were qualified for quantification (Figure 2). Overall 1,297 phosphosites were significantly altered due to TcdB or TcdB_NXN_ treatment (2 nM for 8 h) (Figure 4). Interestingly, the glucosyltransferase deficient TcdB_NXN_ had a strong impact on the phosphoproteome. These effects should be independent from inactivation of small GTPases by glucosylation. In previous proteome studies a strong input on proteome homeostasis has been observed for TcdB_NXN_ treated cells indicating glucosyltranferase-independent activities of TcdB (Erdmann et al., 2017). Glucosyltransferase active TcdB induces, as expected, cell rounding due to cytoskeleton rearrangement in about 80% of Hep-2 cells and a pyknotic morphology; in the remaining 20% of cells.

All significantly regulated phosphopeptides were compared and many showed a consensus sequence of a clearly proline-dependent phosphorylation sites (S/T-P), which are the target motifs of MAP and cyclin-dependent kinases (Bobo et al., 2013). This correlates well with earlier observations in which it has been reported that apoptosis induced by TcdB is mediated by MAPK-dependent caspase activation (Trost et al., 2009).

The treatment with the enzymatically active TcdB led to generally stronger regulated phosphosites with higher fold changes (Figure 4B and Tables 1, 2). Interestingly, the majority of altered p-sites were up-regulated indicating an activation of signaling transduction pathways as a result of treatment with a glucosyltransferase-active toxin. When looking at the top 10 up- and down-regulated proteins it is noticeable that the phosphorylation status of many GTPase effector proteins and GEFs such as Cdc42 effector protein 1/4 and Arhgef2 and Dock5 were altered after TcdB treatment. As expected this was not the case for TcdB_NXN_. Also AP-1 transcription factor phosphorylation sites were highly phosphorylated, which is a downstream factor of p38 and JNK signaling pathways and is activated as a result of stress and apoptosis (Nateri et al., 2004; Wang et al., 2007). In case of TcdB_NXN_ treatment many cytoskeleton and cell proliferation regulators like PPP1R12A and SRSF2 phosphorylation sites were up-regulated. The SRSF2 protein is known to be phosphorylated in response to apoptosis (Edmond et al., 2011). The 40S ribosomal protein S6 phosphopeptides were strongly downregulated (Table 2). This ribosomal subunit is involved in cell growth and proliferation by mTOR signaling pathway and is dephosphorylated at growth arrest (Magnuson et al., 2012).

**Table 2:** Top 10 up- and down-regulated phosphosites after 8 h treatment with TcdB_NXN_.

Uniprot ID	Gene name	Protein name	Peptide sequence	log_2_ Ratio TcdB_NXN_/control	*p*-Value	Amino acid	Peptide score
Q01130	SRSF2	Serine/arginine-rich splicing factor 2	SK**S**PPKSPEEEGAVSS	3.27	2.84E-03	S-208	93.196
O14974	PPP1R12A	Protein phosphatase 1 regulatory subunit 12A	RLA**S**TSDIEEK	2.94	2.33E-03	S-507	142.95
Q09666	AHNAK	Neuroblast differentiation-associated protein AHNAK	LK**S**EDGVEGDLGETQSR	2.92	2.85E-03	S-135	255.63
Q8WX93	PALLD	Palladin	IA**S**DEEIQGTK	2.86	6.28E-03	S-893	181.75
O94906	PRPF6	Pre-mRNA-processing factor 6	LSQVSDSVSGQ**T**VVDPK	2.85	1.53E-02	T-266	143.47
P16989	YBX3	Y-box-binding protein 3	**S**VGDGETVEFDVVEGEK	2.75	1.18E-02	S-102	149.44
Q8IVT2	MISP	Mitotic interactor and substrate of PLK1	HL**S**ESSGKPLSTK	2.75	3.82E-02	S-471	144.41
G5E9C8	SOS1	Son of sevenless homolog 1	SA**S**VSSISLTK	2.57	3.82E-02	S-1119	81.789
E9PFD7	EGFR	Receptor protein-tyrosine kinase	ELVEPL**T**PSGEAPNQALLR	2.36	1.26E-03	T-648	218.81
P29966	MARCKS	Myristoylated alanine-rich C-kinase substrate	GEPAAAAAPEAGA**S**PVEK	2.31	1.05E-03	S-101	185.3
P62753	RPS6	40S ribosomal protein S6	LS**S**LRAS**T**SKSESSQK	−2.39	1.84E-03	S-236;T-241	112.12
P62753	RPS6	40S ribosomal protein S6	RL**SS**LR	−2.23	5.54E-04	S-235;S-236	126.16
P62753	RPS6	40S ribosomal protein S6	RL**S**SLR	−1.97	4.04E-03	S-235	126.16
P62753	RPS6	40S ribosomal protein S6	RLS**S**LR	−1.93	3.41E-02	S-236	126.16
P62753	RPS6	40S ribosomal protein S6	RLSSLRA**S**TSK	−1.86	5.35E-03	S-240	125.84
Q9UQ35	SRRM2	Serine/arginine repetitive matrix protein 2	SSRS**S**PELTRK	−1.71	3.25E-03	S-1694	110.55
A0A0A0MT60	FKBP15	Peptidyl-prolyl cis-trans isomerase	SSL**S**GDEEDELFK	−1.69	6.89E-03	S-1189	175.27
G3V160	CNKSR1	Connector enhancer of kinase suppressor of ras 1	SPSLSLAPL**S**PR	−1.62	1.26E-02	S-49	118.2
Q92797	SYMPK	Symplekin	SPQTLAPVGE DAMKTP**S**PAAEDAREPEAK	−1.62	2.73E-03	S-1259	111.09
A0A096LNZ0	AAK1	Uncharacterized protein FLJ45252	LGGAVPFAPPEV**S**PEQAK	−1.58	5.82E-04	S-217	130.37

Phosphosites with MAP kinase motifs were differently regulated in TcdB and TcdBNXN. These kinases are particular involved in ERK, JNK und p38 signaling pathways (Pearson et al., 2001). Several proteins belonging to these pathways have been identified in a proteome analysis to be regulated in similar treated cells (Junemann et al., 2016). Particularly RAF activators and transcription factors, e.g., AP-1 localized downstream of JNK signaling are involved. These pathways regulates apoptotic processes and cell cycle arrest (Dhanasekaran and Reddy, 2008, Huang et al., 2009). These hypothesis are supported by further studies that provide evidence for a caspase-mediated apoptosis induced by C. difficile toxins (Qa’Dan et al., 2002; Solomon et al., 2005; Nottrott et al., 2007). First an activation of inhibitory caspases 8 and 9 is induced with an subsequent activation of known effector caspases 3, 6, and 7 that are involved in the cytotoxic effects induced by C. difficile toxins.

At a first glance these preliminary results suggest signaling pathways to be affected by TcdB or TcdB_NXN_ treatment. Nevertheless, further investigations are necessary to further elaborate and confirm these findings.

## Author Contributions

AP conceived the idea. JJ performed the experiments. JJ, AP, and RG did data analyses. JJ, RG, IJ, and AP wrote the manuscript and approved the final version.

## Conflict of Interest Statement

The authors declare that the research was conducted in the absence of any commercial or financial relationships that could be construed as a potential conflict of interest.

## Supplementary Material

TABLE S1 Identified proteins.

TABLE S2 Identified phosphopeptides.
